# The Impact of Heterogeneity in Individual Characteristics and Motivation to Quit Heroin Use on Contingency Management and Opioid Agonist Treatment Response: A Latent Class Analysis

**DOI:** 10.1111/dar.70264

**Published:** 2026-10-07

**Authors:** Louise MacGregor, Timothy Weaver, John Strang, Stephen Pilling, Nicola Metrebian, Kimberley Goldsmith

**Affiliations:** ^1^ Department of Biostatistics and Health Informatics Institute of Psychiatry, Psychology and Neuroscience, King's College London London UK; ^2^ Department of Mental Health & Social Work Middlesex University London UK; ^3^ National Addiction Centre Institute of Psychiatry, Psychology and Neuroscience, King's College London London UK; ^4^ Clinical, Educational and Health Psychology Department University College London London UK

**Keywords:** contingency management, individual differences, latent class analysis, opioid use disorder, precision care

## Abstract

**Introduction:**

Contingency Management (CM) involves the systematic application of positive reinforcement (financial incentives) to promote behaviour change, to improve treatment outcomes for individuals with opioid use disorder (OUD). To better understand patient heterogeneity, this study sought to identify meaningful subgroups/classes underlying the population based on characteristic groupings, followed by determining whether CM effectiveness varied between these subgroups.

**Methods:**

Baseline characteristics (evidenced/theorised to impact CM effectiveness) of participants with OUD from the PRAISe trial (*N* = 552) were empirically examined using latent class analysis. Model selection was based on fit statistics and interpretability. Class was then evaluated as a potential moderator of CM effectiveness on abstinence from heroin using a mixed effects model (consistent with the PRAISe trial analysis).

**Results:**

A four‐class solution best described the population: (1) Less clinically complex/low alcohol dependence/high motivation to quit heroin (*N* = 115); (2) More clinically complex/low alcohol/high motivation (*N* = 287); (3) More clinically complex/high alcohol/high motivation (*N* = 73); and (4) More clinically complex/moderate alcohol/low motivation (*N* = 77). CM for Attendance, previously shown to increase heroin abstinence in the overall study sample, was more effective than no CM for participants in class (1) and (2) exclusively (OR = 5.21; 95% CI 1.25, 21.78 and OR = 1.87; 95% CI 1.03, 3.40, respectively). CM targeted at abstinence, previously shown to not be effective compared to no CM overall, was not effective in any class.

**Discussion and Conclusions:**

CM targeted at attendance is more effective for two subgroups of the OUD population characterised by either less complex clinical presentation and/or low dependence on alcohol. Individual differences may therefore inform a targeted CM treatment approach.

## Key Point Summary

- People with opioid use disorder could be classified into four distinct latent classes.
- The effect of Contingency Management (CM) targeted at attendance on heroin abstinence differs across classes.
- CM Attendance was more effective at increasing abstinence for the class characterised by less clinical complexity.
- There was no evidence of the effectiveness of CM Abstinence on heroin abstinence in any of the classes.
- Has potential to guide personalised care and supports a tailored treatment approach.

## 1 Introduction

Opioid use disorder (OUD), particularly heroin use, is a persisting issue across the United Kingdom, with approximately 260,000 users [1]. OUD carries a substantial health burden due to its contribution to the increased risk of physical, mental health and social problems, including the risk of overdose; across England and Wales almost half of drug‐related deaths involve an opiate [2]. Opioid use also has a significant economic impact—86% of the United Kingdom's £19.3 billion annual drug misuse cost is attributable to health and crime‐related costs of heroin and crack cocaine [1].

According to UK clinical guidelines, treatment for OUD should integrate support for pharmacological interventions and psychosocial interventions [3]. The primary pharmacological treatment for OUD is opioid agonist treatment (OAT), a substitution regimen of synthetic opioid agonist medication such as buprenorphine or methadone that helps to prevent withdrawal symptoms and reduce craving. OAT is recognised globally as both clinically and cost‐effective and was used to treat 99% of adults receiving treatment for OUD in England in 2022/23 [2, 3, 4]. Treatment for drug use should also include a psychosocial component including regular sessions with a key individual or practitioner known as a keyworker, responsible for the ongoing review of the care/treatment plan, focused on achieving patient goals. Keyworkers can also deliver some or all the psychosocial elements of care [3, 5].

However, treatment suffers from high attrition rates and patients relapsing into illicit street‐drug use [4], and hence the full benefits, particularly of OAT, have not been realised. The impact of OAT on treatment outcomes also varies dependent on certain individual characteristics. For example, a longer history of pre‐OAT treatment opiate use and pre‐treatment cocaine use/heroin injection reported within the last 30 days are associated with poorer retention [6, 7]. Additionally, prior treatment for opiate addiction, short length of treatment, low use of alcohol and other factors dependent on sex are associated with continued drug use post‐treatment [8, 9].

Contingency Management (CM) is recommended by both UK Clinical Guidelines [3] and the National Institute for Health and Care Excellence (NICE) [10], to be delivered as a psychological element to treatment, alongside OAT. CM is a behavioural intervention based on operant conditioning; whereby positive incentives (material or financial rewards) are administered to service users contingent on achievement of positive behaviour change (e.g., abstinence from street drugs). CM has a strong evidence‐base, primarily from research conducted in the United States, supporting its effectiveness at improving abstinence outcomes from a range of substances including illicit opiates [11, 12, 13, 14]. Most evidence additionally supports the synergistic benefit of delivering CM targeted at opiate abstinence alongside OAT, on abstinence [11]. Through CM increasing abstinence during OAT, CM both directly addresses high relapse rates and subsequent dropout, improving engagement with (and attendance to) treatment [14]. CM can also be targeted towards other treatment goals such as attendance (irrespective of abstinence). CM targeting attendance is both evidenced to improve attendance (of importance due to the high dropout rate of OAT), in addition to having a small effect on improving abstinence, even when abstinence was not the direct behaviour targeted [13].

However, there remains a significant gap between research evidence and practice, with minimal implementation of CM in the United Kingdom and evidence of its effectiveness in a UK National Health Service (NHS) setting (despite recommendation from NICE since 2007). CM (particularly targeted at abstinence) is a resource‐heavy intervention, and the UK drug treatment service has considerable differences from the United States, which likely restrict implementation, including limited treatment budgets and staff capacity, and less experience of providing CM [15]. Previous UK evidence did demonstrate that CM targeted at attendance to vaccination appointments promoted adherence to and completion of a hepatitis B vaccination schedule [16].

A more complex CM intervention model, adapted for UK and NHS settings, investigated the clinical and cost effectiveness of CM on promoting heroin abstinence through a UK cluster randomised controlled trial (PRAISe). The PRAISe trial compared CM schedules over 12 weeks targeted at: (i) Abstinence (reinforcement conditional on attendance at weekly clinical appointments and provision of opiate‐negative urine drug screens (UDS) (reinforcement given at 1–4 weeks irrespective of UDS result)); (ii) Attendance (on‐time attendance to weekly clinical appointments only); and (iii) No CM/TAU (treatment as usual, OAT plus 12× weekly appointments), for patients seeking a new episode of OAT [17]. The main comparisons of interest were comparisons with TAU. CM Attendance was included to determine whether heroin abstinence could be derived from increased attendance at clinical appointments.

This trial concluded that CM Attendance was moderately effective at encouraging heroin‐abstinence compared to TAU, whereas CM Abstinence was not more effective than TAU or CM Attendance at 12 weeks [18]. Findings aligned with previous research on CM targeted at attendance [13]. However, the lack of effectiveness of CM targeted at Abstinence does not align with previous research [11, 13], and was regarded by the authors as a potential result of adaptations made to the CM model to fit the UK/NHS system; for example, fixed incentives rather than escalating incentives and a once‐weekly UDS instead of multiple tests per week [18].

Previous evidence has supported the efficacy of CM (targeting abstinence) for patients with substance use disorders (including OUD), regardless of patients' baseline characteristics or pre‐existing conditions [15]. However, a recent systematic review found evidence that specific patient‐level characteristics including baseline patient demographics, drug/alcohol treatment history and motivation to stop substance use (of individuals using opioids and/or stimulants and accessing drug and alcohol outpatient services predominantly in the United States) are associated with either enhanced or diminished response to CM [19]. The PRAISe study investigated the effectiveness of CM (at improving abstinence only) for a somewhat heterogenous population of individuals presenting for OAT at drug and alcohol services in England; likely including individuals with different personal treatment objectives and also differing responses to CM.

The systematic review by Forster et al. [19] had limitations; patient characteristics were investigated individually, whereas in actuality patients are heterogeneous across multiple characteristics simultaneously. This heterogeneity could be thought of as manifesting in ‘symptom/characteristic groupings’, where a range of these aspects come together to describe subgroups of interest. Latent class analysis (LCA) is a statistical approach used to explore this heterogeneity, through identifying qualitatively different subgroups underlying the population across a range of observed characteristics known as indicators [20]. As the field of precision medicine continues to advance, the potential applications of LCA are promising; particularly in evaluating treatment effectiveness through identifying subpopulations of patients most likely to respond to a particular treatment [21]. Much as Van Der Stel has alluded to, this could facilitate the development in addiction care for individualised medicine, through targeted treatment strategies [22]. It could be the case that having a certain cluster of characteristics may moderate the effectiveness of CM for the patient. Following this, CM (attendance‐based or abstinence‐based) could be targeted to those most likely to benefit, improving individual patient outcomes. As resources and funding are a significant setback to CM implementation in the United Kingdom, strategically targeting CM could increase benefits at reduced cost.

This study therefore used LCA to determine if meaningful classes exist within the PRAISE trial population characterised by baseline variables theorised/evidenced to individually impact CM effectiveness versus TAU (Aim 1) and to determine if the class variable modified the effectiveness of CM vs. TAU (Aim 2). Though participants were enrolled on this trial between 2012 and 15, the population in treatment for OUD today remains similar within the United Kingdom [2] and therefore the findings are generalisable to the current population and situation.

## 2 Methods

### 2.1 Study Design

This study was a secondary data analysis of an existing dataset derived from a multi‐site UK cluster randomised controlled trial (PRAISe). Participants were enrolled in sites/clusters randomly allocated to receive OAT plus 12× weekly keyworker appointments with either: (i) CM targeted at attendance at clinical appointments and opiate abstinence; (ii) CM targeted at on‐time attendance only; or (iii) no CM (TAU), more details of which are in the trial protocol paper [17]. A latent class analysis was used to determine groupings of characteristics underlying the trial population, characterised by baseline characteristics theorised/evidenced to impact CM effectiveness [19]. Subgroup was then evaluated as a potential moderator of CM effectiveness (targeted at attendance or abstinence) within the PRAISe trial.

The PRAISe trial received research ethics approval from NRES Committee South East Coast Surrey (12/LO/0910) and Trial registration number: ISRCTN 01591254.

### 2.2 Participants

Participants were individuals with OUD regularly using street heroin (self‐reported use 15/preceding 30 days, at least 3‐days/week and all UDS in previous month positive for opiates), presenting for OAT (methadone or buprenorphine), and enrolled across 33 NHS and non‐NHS drug treatment clinics in England between November 2012 and October 2015 (*n* = 552), see trial paper for further inclusion/exclusion criteria [18].

### 2.3 Measures

Baseline measures used as indicator variables in the latent class analysis are outlined in Sections 2.3.1, 2.3.6. The response/urine test outcome measure (PRAISe trial primary outcome) is described in Section 2.3.7.

#### 2.3.1 Sociodemographic Measures

Sociodemographic measures used were age, sex (male/female), ethnicity (White/other) and living situation categorised as homeless/not homeless.

#### 2.3.2 Opiate and Other Illicit Drug Use Measures

Opiate and other drug use measures include age participant first used opiates, whether the participant had ever injected opiates (yes/no), self‐reported use of crack cocaine in the last 30 days (yes/no) and/or self‐reported use of benzodiazepine in the last 30 days (yes/no) from the Opiate Treatment Index drug use questionnaire [23, 24], and treatment history measured via the number of previous treatment attempts (< 2 or ≥ 2 previous treatment attempts, as Forster et al. used in [19]).

#### 2.3.3 Alcohol Use Measure

The total Alcohol Use Disorders Identification Test (AUDIT) score was used [25], a 10‐item questionnaire used as a screening instrument for alcohol dependence. Responses to each question are scored from 0 to 4 with a sum AUDIT score of up to 40, and a higher score indicating higher risk for alcohol dependence. The AUDIT score has good psychometric properties; a median reliability coefficient of 0.83, high test–retest reliability and high internal consistency [25, 26].

#### 2.3.4 Mental Health Subscale

The Mental Health component summary of the Short Form‐36 version II [27], composed of eight multi‐item scales assessing functional health and well‐being from the perspective of the patient and combined into the Mental Health component summary using norm‐based scoring (with a mean of 50 and standard deviation of 10) was used. A higher score indicates a more favourable mental health state. This scale has shown high validity and reliability [28, 29].

#### 2.3.5 Drug Use Motivation Subscales

A scale with intrinsic face validity was constructed for use in PRAISe to assess intention to quit heroin use. This was scored on a scale of 1–10 through obtaining the average of four questions looking at readiness, confidence, commitment and importance of reducing heroin use. Higher scores indicate higher motivation.

#### 2.3.6 Impulsivity Measure

A binary impulsivity measure (delay discounting) was used, which describes how quickly incentives decrease in value to an individual as the time before receiving the incentive increases. ‘High’ delay‐discounting (choosing smaller rewards sooner) is associated with relevant behaviours related to substance use disorder [30, 31, 32, 33]. Participants chose either immediate consumption of a smaller hypothetical monetary reward (£45 available after 3 days) or delayed consumption of a larger hypothetical monetary reward (£70 available after 3 months [34]).

#### 2.3.7 Distal Outcome

The binary PRAISe trial primary outcome of a once‐weekly binary positive/negative heroin UDS collected at weeks 9–12 post randomisation was used as the distal outcome. Refusal to provide a urine sample/non‐attendance was coded as positive (the positive assumption commonly used in clinical practice and in studies of CM and was used in our previous trial [35]), but results missing for other reasons such as discharged from treatment, in hospital or in prison were left missing, as in the original PRAISE trial [18].

### 2.4 Descriptive Statistics

Descriptive statistics of indicators are presented, including counts and percentages, means and SDs, and medians and interquartile ranges as appropriate, along with number of missing observations. Some of these summary statistics that were reported in the original PRAISE trial paper [18] are reported again here for completeness.

### 2.5 Analysis

For analysis, indicators with skewed continuous distributions were normalised using a log‐transform (if effective). Categorical variables with low frequencies (< 10%) that have limited distributional information were collapsed into larger categories (as described in Section 2.3). All continuous variables (and logged‐continuous variables) were *Z*‐standardised.

A 3‐step LCA procedure was implemented using MPlus [36]: Step 1: a standard latent class model was estimated using only indicator variables; Step 2: patients were assigned to their most likely class and the measurement error for the class variable was determined; Step 3: the most likely class was used as a moderator in the outcome model (with measurement error for class variables fixed and pre‐specified to the values determined in Step 2 [37]).

In Step 1, classes underlying the trial population were identified using all indicator variables listed excluding the distal outcome. LCA was performed using an iterative process starting with a one‐class solution, then models of one additional class at a time were specified up to 6 classes. Models were estimated using Full‐information Maximum Likelihood and identified using 100 random starting values with 50 iterations.

Selection of the optimal number of classes was based on the model Bayesian information criterion (BIC) [38], sample‐size adjusted BIC (SS‐BIC) [39], Akaike's information criterion [39, 40], the Lo–Mendell–Rubin Likelihood Ratio test [41], smallest class size (%), entropy and model interpretability. Lower values of the BIC, Akaike's information criterion and SS‐BIC indicate a better balance between model fit and parsimony; the Lo–Mendell–Rubin Likelihood Ratio test can be used to determine whether the improvement in fit between *k* − 1 and *k* class models is statistically significant; a small class size (< 5% N) can indicate over‐fitting and lack of generalisability; and a higher value for entropy indicates more precise assignment of individuals to a latent class and hence better class separation. Though there is no strong consensus in the literature regarding the best criteria for comparing latent class solutions, the lowest BIC and SS‐BIC are generally regarded as the best performing indices [42, 43]. Interpretability was assessed through inspecting the distributions of indicator values for each class, with clinical input. Considering all factors, a final latent class model was chosen.

The distribution of indicator variables across the classes (the de‐standardised marginal means of continuous variables and probability of endorsement of categorical variables) in the optimal class model is presented, and compared between classes using the chi‐square test, analysis of variance and the Tukey multiple comparisons procedure, and the Kruskal–Wallis non‐parametric test.

For Step 3, a two‐level logistic mixed effect regression model was fitted; the distal outcomes of repeated weekly measures of binary negative heroin UDS (yes/no) at weeks 9–12 were treated as the dependent variables with time as a Level 1 variable, and trial arm, clinic type stratification factor (NHS vs. non‐NHS) at Level 2 (participant), with the effect size allowed to vary by latent class variable (the most likely class variable for each participant), and the class measurement error fixed and prespecified to the values computed in Step 2 to account for uncertainty in the class assignments. The Huber sandwich estimator was used to compute robust standard errors to account for variation due to clustering within site. The odds ratio estimate of negative UDS for CM Abstinence versus TAU and CM Attendance versus TAU in the PRAISe trial were estimated for each level of the moderator (class).

## 3 Results

Descriptive statistics of the whole PRAISe population (see Section 2.2 for Inclusion criteria) for all 13 baseline characteristics selected as indicator variables are presented in Table 1. The average participant age was 38 (SE = 8.8) and participants were mostly male (73.2%) and White (79.5%). A significant proportion of participants were homeless (31.6%) and had been in treatment for OUD on two or more previous occasions (59.3%). The reported mean age of first opiate use was 23.4 (SE = 7.7). Just over half of participants had reported ever injecting opiates (59.7%). Most participants were using crack cocaine in the 30 days before baseline (75.9%) and most participants were not using benzodiazepines within the 30 days before baseline (23.4%). Most participants opted for a more immediate, smaller reward with regards to delay discounting (80.2%), indicating high impulsivity. The median AUDIT score was 6 out of 40 (1–15), indicating low‐risk alcohol consumption, and the mean SF‐36 mental health score was 30.8 (SE = 14.4) on a norm‐based scoring scale, indicating poor mental health. Participants reported being strongly motivated to quit heroin use altogether, represented by a median score of 9 (8–10) out of 10.

**TABLE 1:** Descriptive statistics of baseline variables used as indicators.

	Summary	Missing/*N*
Sociodemographics		
Age, years, mean (SD)	38.18 (8.83)	0/552
Gender, *N* (%)		0/552
Male	404 (73.2%)	
Female	148 (26.8%)	
Ethnicity^a^, *N* (%)		5/552
White	435 (79.5%)	
Black	33 (6.0%)	
Asian	42 (7.7%)	
Mixed	37 (6.8%)	
Homelessness^b^, *N* (%)		1/552
Not homeless	377 (68.4%)	
Homeless	174 (31.6%)	
Number of previous opiate treatment attempts, *N* (%)		11/552
< 2	220 (40.7%)	
≥ 2	321 (59.3%)	
Age first used opiates (years), mean (SD)	23.46 (7.72)	10/552
Ever injected opiates, *N* (%)		14/552
No	217 (40.3%)	
Yes	321 (59.7%)	
Crack cocaine use in past 30 days, *N* (%)		0/552
No	133 (24.1%)	
Yes	419 (75.9%)	
Benzodiazepine use in past 30 days, *N* (%)		1/552
No	422 (76.6%)	
Yes	129 (23.4%)	
Total AUDIT score, median (LQ‐UQ)	6 (1–15)	0/552
Delay discounting measure, *N* (%)		1/552
£45 in 3 days time	442 (80.2%)	
£70 in 3 months time	109 (19.8%)	
SF36 Mental Health Subscale, mean (SD)	30.75 (14.36)	2/552
Motivation to quit heroin use, median (LQ‐UQ)	9 (8–10)	2/552

*Note:* Total sample: *N* = 552. For all continuous variables; mean (standard deviation = SD) given. For all categorical variables; *N* (%) given ‐ all participants missing data on each indicator are excluded from percentage descriptions of categories; this differs from how these demographics were presented in the original PRAISe trial. For all skewed continuous variables; median (25th percentile = LQ; 75th percentile = UQ) given.

Abbreviation: AUDIT, Alcohol Use Disorders Identification Test.

^a^ *N* (%) shown for all ethnicity categories recorded, however, variable is recoded as binary for use as an indicator (White or Other only).

^b^ Participants are classed as homeless if they are one of the following: living with parents/relatives, in a B&B/hotel, hostel, no fixed address (living on streets) or house sitting. Participants are classed as not homeless if they are an owner/occupier, live in rented private accommodation or rented (local authority/housing association).

Model fit and selection criteria information for the latent class models are presented in Table 2. All latent class models had entropy values > 0.8 indicating good class separation. The BIC was lowest for the 5‐class model (12744.7), followed by the 4‐class model (12759.5). The Lo‐Mendel Rubin log‐likelihood test indicated the 4‐class model was the most parsimonious (5‐class model not significantly better than the 4‐class model, *p* = 0.230). Taking into consideration the smallest latent class size, which decreased significantly from four classes to five classes (from 13.2% to 2.4%) possibly indicating overfitting and affecting interpretability, the 4‐class model was chosen as the optimal model.

**TABLE 2:** Fit indices for class solutions.

No. of classes	LL	df	LMR‐LL	AIC	BIC	SS‐adjusted BIC	Smallest class (% *N*)	Entropy
1	−6442.8	18	N/A	12921.5	12999.1	12942.0	N/A	N/A
2	−6327.7	32	227.6 (*p* < 0.001)	12719.4	12857.4	12755.8	18.3	0.88
3	−6251.5	46	150.7 (*p* = 0.035)	12594.9	12793.4	12647.3	13.6	0.89
4	−6190.4	60	120.9 (*p* < 0.001)	12500.7	12759.5	12569.1	13.2	0.82
5	−6138.8	74	102.0 (*p* = 0.230)	12425.5	12744.7	12509.8	2.4	0.85
6	−6113.7	88	49.5 (*p* = 0.425)	12403.5	12783.1	12503.7	2.2	0.80

*Note:* Smallest class based on most likely class membership.

Abbreviations: AIC, Akaike's information criterion; BIC, Bayesian information criterion; LL, log‐likelihood; LMR‐LL, Lo–Mendel–Rubin log‐likelihood; N/A, not applicable; SS‐adjusted BIC, sample‐size adjusted Bayesian information criterion.

The individuals in the class we describe as ‘Less complex clinical presentation, low risk of alcohol dependence and high motivation to quit heroin’, representing 20.8% of the population, had: never injected opiates, significantly less previous treatment attempts, better mental health, lower levels of homelessness, and less past month benzodiazepine and crack cocaine use relative to all other subgroups (see Table 3; Figure 1). The age of onset of opiate use was also later, and this population had lower AUDIT scores indicating low risk of alcohol dependence, high motivation to quit heroin use and a higher proportion of ethnic minority groups (Black, Asian or Mixed) than the other subgroups, who were predominantly White.

**TABLE 3:** Marginal probabilities and means of indicators (with 95% CI) across each class of the 4‐class model.

	Less complex clinical presentation, low risk of alcohol dependence and high motivation to quit heroin, *N* = 115 (20.8%)	More complex clinical presentation, low risk of alcohol dependence and high motivation to quit heroin, *N* = 287 (52.0%)	More complex clinical presentation, high risk of alcohol dependence and high motivation to quit heroin, *N* = 73 (13.2%)	More complex clinical presentation, low/moderate risk of alcohol dependence and low motivation to quit heroin, *N* = 77 (13.9%)	Test (d.f. = 3), *p*
Categorical indicators					
% Male	79.7 (67.3–92.1)	70.8 (64.9–76.8)	65.3 (53.4–77.1)	81.3 (71.8–90.9)	*χ* ^2^ = 5.4 (*p* = 0.143)
% Homeless	16.3 (6.5–26.2)	34.6 (28.4–40.8)	40.0 (27.9–52.0)	32.3 (18.8–45.8)	*χ* ^2^ = 18.3 (*p* < 0.001)
% Crack cocaine use in past 30 days (yes)	67.2 (56.0–78.4)	75.6 (70.2–81.0)	80.0 (69.8–90.2)	84.6 (75.2–93.9)	*χ* ^2^ = 10.3 (*p* = 0.016)
% Benzodiazepine use in past 30 days (yes)	5.1 (−3.6–13.9)	24.6 (19.2–30.1)	32.2 (20.5–43.8)	34.7 (22.1–47.2)	*χ* ^2^ = 31.9 (*p* < 0.001)
% £70 in 3 months time vs. £45 in 3 days time	17.4 (8.0–26.9)	24.2 (18.7–29.6)	17.9 (8.3–27.6)	8.1 (1.3–14.8)	*χ* ^2^ = 12.4 (*p* = 0.006)
% ≥ 2 previous treatment attempts	35.4 (22.9–47.9)	66.5 (60.0–73.0)	51.7 (38.4–65.1)	71.7 (60.5–82.9)	*χ* ^2^ = 50.8 (*p* < 0.001)
% Ethnicity White (vs. other)	47.6 (32.2–62.9)	89.7 (85.3–94.0)	86.6 (77.6–95.6)	77.3 (67.1–87.6)	*χ* ^2^ = 98.9 (*p* < 0.001)
% Have ever injected opiates	0.00 (0–0)	77.4 (67.1–87.7)	60.2 (46.9–73.5)	70.6 (58.8–82.4)	*χ* ^2^ = 212.6 (*p* < 0.001)
Continuous indicators (normally distributed)					
Age, years	38.8 (36.4–41.1)	37.2 (36.0–38.4)	38.1 (35.9–40.3)	41.2 (38.7–43.8)	*F* = 1.9 (*p* = 0.601)
Age (years) of first using opiates	27.0 (25.0–29.1)	20.8 (19.9–21.7)	22.5 (20.5–24.7)	22.5 (20.8–24.4)	*F* = 3.5 (*p* = 0.316)
SF‐36 Mental Health Subscale	35.5 (30.9–40.1)	30.2 (28.3–32.0)	28.7 (25.6–31.8)	28.5 (25.2–31.8)	*F* = 9.0 (*p* = 0.029)
Skewed continuous indicators					
AUDIT score	4.7 (3.3–6.0)	5.7 (4.9–6.6)	27.9 (25.9–30.0)	11.4 (8.5–14.3)	*H* = 195.4 (*p* < 0.001)
Motivation to quit heroin use	9.2 (9.0–9.4)	9.0 (8.9–9.2)	8.8 (8.6–9.1)	5.7 (5.2–6.1)	*H* = 169.1 (*p* < 0.001)

*Note: n* and %*N* for each class are presented. For categorical indicators, marginal proportions are presented for each class with the results of the chi‐square statistical test for assigned class values. For normally‐distributed continuous indicators, marginal means are presented for each class with the results of a one‐way analysis of variance test for assigned class values. For skewed continuous indicators, marginal means are presented for each class and the results of the Kruskal–Wallis test for assigned class values.

Abbreviations: AUDIT, Alcohol Use Disorders Identification Test; CI, confidence interval.

**FIGURE 1:**
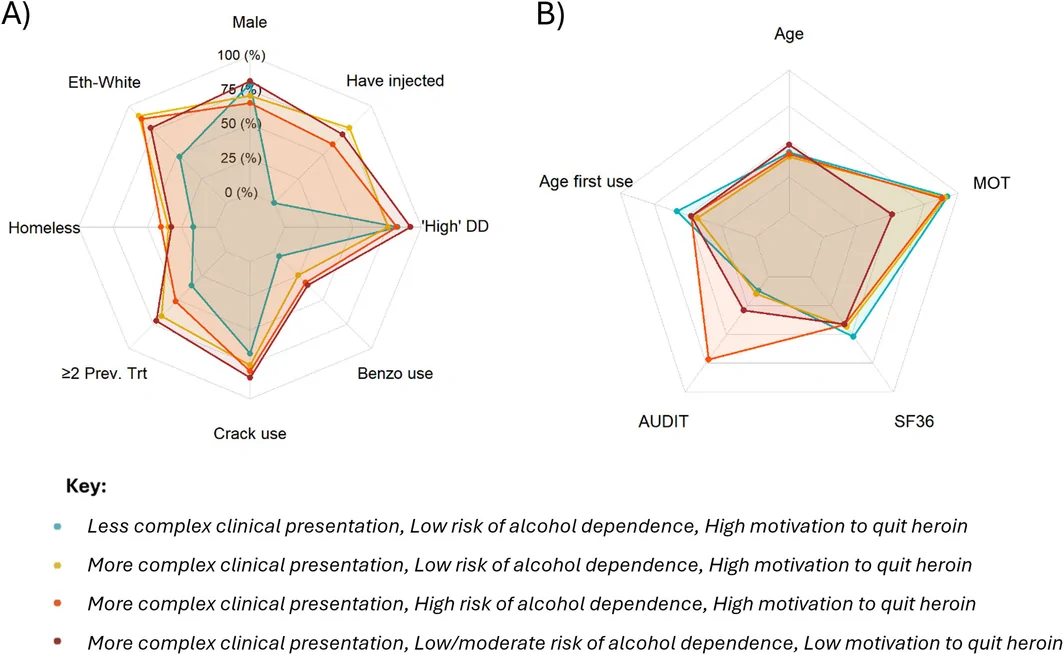
Radar plots representing the four‐class model. Categorical and continuous indicators represented separately. (A) The marginal proportion of endorsement for each categorical indicator across classes of the four‐class model. (B) The marginal *z*‐standardised means for each continuous indicator across classes of four‐class model. Have injected, proportion who have reported injecting opioids; ‘High’ DD, ‘high’ delay discounting—proportion that chose £45 in 3 days time instead of £70 in 3 months time; Benzo use/Crack use, proportion of population recording crack cocaine or benzodiazepine use in the past 30 days; ≥ 2 Prev. Trt, proportion having two or more previous opiate treatment attempts; Homeless, proportion living with parents/relatives, in a B&B/hotel, hostel, no fixed address (living on streets) or house sitting; Eth‐White, proportion of population of White ethnicity; MOT, motivation to quit heroin use; SF36, SF‐36 Mental Health Subscale; AUDIT, Alcohol Use Disorders Identification Test; Age first use, the standardised log‐transform of age first used opiate.

All three other subgroups have ‘More Complex’ clinical presentation characterised by a significantly higher proportion of participants who have reported ever injecting opiates, homelessness, a greater number of previous treatment attempts, past‐month crack cocaine and benzodiazepine use and worse mental health scores. We describe the largest class, representing 52% of the population, as ‘More complex clinical presentation, low risk of alcohol dependence and high motivation to quit heroin’. In addition to ‘More Complex’ clinical presentation (with some variation such as significantly less past month benzodiazepine use than other ‘More Complex’ subgroups), individuals in this class reported a low risk of alcohol dependence and high motivation to quit heroin use. For the third class (13.2% of the population), ‘More complex clinical presentation, high risk of alcohol dependence and high motivation to quit heroin’, these individuals had possible alcohol dependence in addition to ‘More Complex’ clinical presentation (with some variation such as significantly less participants reporting ≥ 2 previous treatment attempts than other ‘More Complex’ subgroups) and high motivation to quit heroin use. The fourth class ‘More complex clinical presentation, low/moderate risk of alcohol dependence and low motivation to quit heroin’, represented 13.9% of the trial population. In addition to ‘More Complex’ clinical presentation, this class was significantly older, reported an increasing risk of alcohol dependence, and had a distinctly lower motivation to quit heroin use, in addition to ‘high’ delay discounting.

### 3.1 Determining Whether the Effectiveness of CM Differed Between Classes

As in the original PRAISE study, 601 of the 2208 scheduled UDS for weeks 9–12 were not obtained due to non‐compliance (refusal or DNA) and were coded as positive on the ‘positive assumption’. Five hundred and one further UDS were coded as missing. Sixty‐nine participants who had been assigned to a class were excluded from the analysis due to missing all four observations at weeks 9, 10, 11 and 12; 34 of those excluded received CM targeting abstinence (16.6% of participants assigned to this arm), 12 (6.9%) received CM targeting attendance and 23 (13.3%) received TAU. From the ‘Less complex clinical presentation, low risk of alcohol dependence and high motivation to quit heroin’ class, 14 (12.3% of N belonging to the class) were excluded, 36 (12.5%) from the ‘More complex clinical presentation, low risk of alcohol dependence and high motivation to quit heroin’ class, 7 (9.6%) from the ‘More complex clinical presentation, high risk of alcohol dependence and high motivation to quit heroin’ class, and 12 (15.6%) from the ‘More complex clinical presentation, low/moderate risk of alcohol dependence and low motivation to quit heroin use’ class. Participants in CM Abstinence attended a mean number of 6.13 (SD 4.03) keywork appointments over the 12 weeks, compared with participants in the CM Attendance group who attended a mean number of 7.45 (3.84) and those in TAU a mean number of 4.80 (3.33).

CM abstinence and CM attendance versus TAU effects on heroin‐negative UDS results across weeks 9–12 are presented in Figure 2 by class. Participants particularly within the ‘*Less* complex clinical presentation, low risk of alcohol dependence and high motivation to quit heroin’ class, and to a lesser extent the ‘*More* complex clinical presentation, low risk of alcohol dependence and high motivation to quit heroin’ class, treated with CM Attendance, had significantly greater odds of a heroin‐negative urine at 9–12 weeks compared with those in the TAU arm (odds ratio [OR] 5.21; 95% confidence interval [CI] 1.25, 21.78 and OR 1.87; 95% CI 1.03, 3.40, respectively). CM Attendance was not significantly more effective than TAU at improving odds of a heroin‐negative urine at 9–12 weeks for either of the other two classes (OR 2.25; 95% CI 0.69, 7.41 and OR 2.61; 95% CI 0.68, 10.06, for the ‘More complex clinical presentation, high risk of alcohol dependence and high motivation to quit heroin’ class, and the ‘More complex clinical presentation, low/moderate risk of alcohol dependence and low motivation to quit heroin’ class, respectively).

**FIGURE 2:**
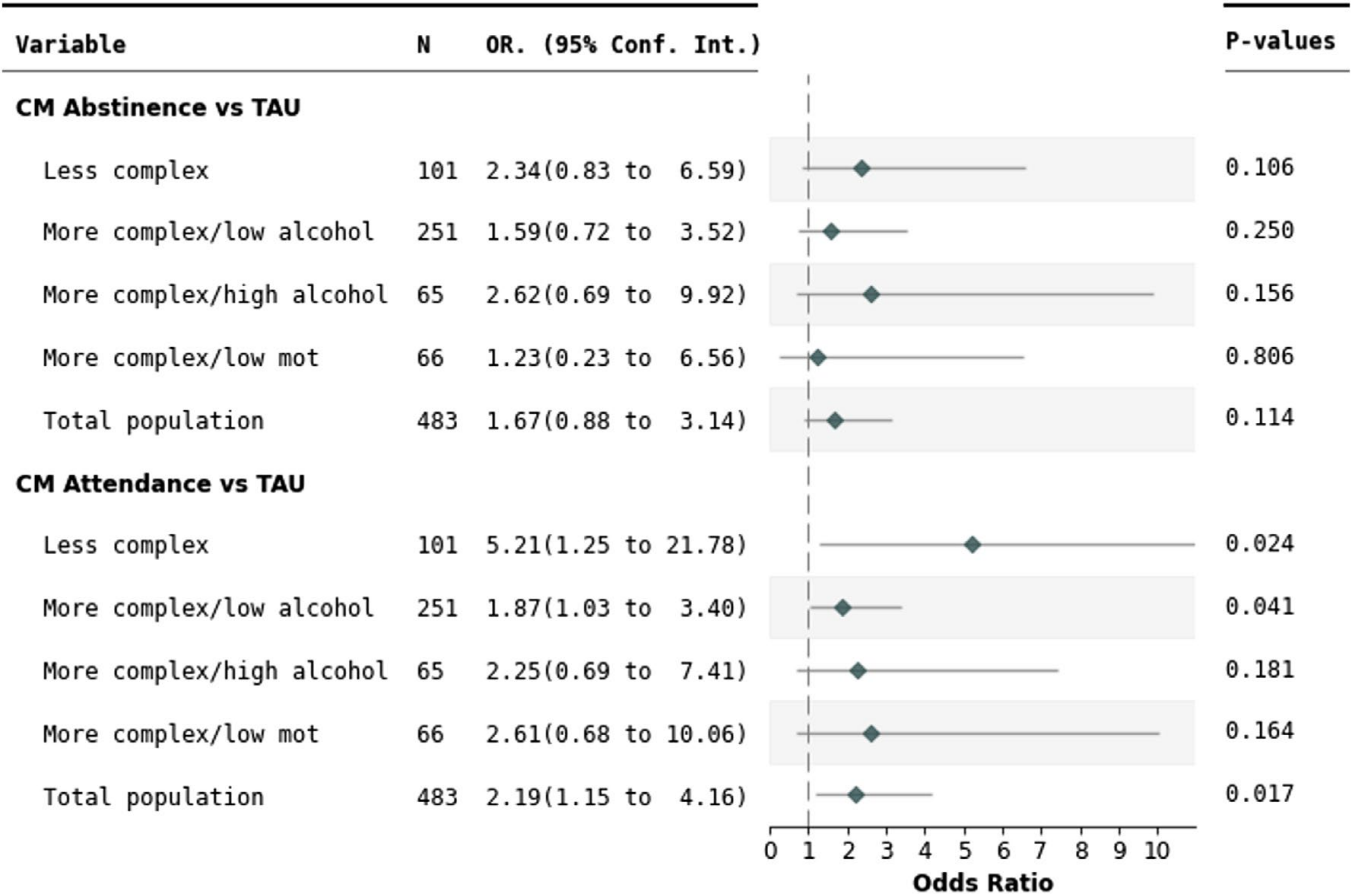
Forest plot of the odds of participants providing a heroin‐negative urine over weeks 9–12 for CM Abstinence and CM Attendance arms (vs. TAU), within each class of the 4‐class model. Odds ratio estimates for *N*; the subset of the trial population within each class included in the model, excluding 69 dropped participants. CM, Contingency Management targeted at; TAU, treatment as usual; Less complex, ‘Less complex clinical presentation, low risk of alcohol dependence and high motivation to quit heroin’ class; More complex/low alcohol, ‘More complex clinical presentation, low risk of alcohol dependence and high motivation to quit heroin’ class; More complex/high alcohol, ‘More complex clinical presentation, high risk of alcohol dependence and high motivation to quit heroin’ class; More complex/low mot, ‘More complex clinical presentation, low/moderate risk of alcohol dependence and low motivation to quit heroin’ class. *Main results of PRAISe trial [18].

CM Abstinence was not significantly more effective than TAU at increasing the odds of a heroin‐negative UDS at weeks 9–12 in any of the four classes; the ‘Less complex clinical presentation, low risk of alcohol dependence and high motivation to quit heroin’ class (OR 2.34; 95% CI 0.83, 6.59), the ‘More complex clinical presentation, low risk of alcohol dependence and high motivation to quit heroin’ class (OR 1.59; 95% CI 0.72, 3.52), the ‘More complex clinical presentation, high risk of alcohol dependence and high motivation to quit heroin’ class (OR 2.62; 95% CI 0.69, 9.92) and the ‘More complex clinical presentation, low/moderate risk of alcohol dependence and low motivation to quit heroin’ class (OR 1.23; 95% CI 0.23, 6.56).

## 4 Discussion

This study sought to identify groupings of patient characteristics among individuals receiving OAT for OUD in a UK‐based randomised controlled trial, PRAISe, and examine how these subgroups responded to OAT and CM. Four distinct patient sub‐groups were identified using latent class analysis; the ‘Less complex clinical presentation, low risk of alcohol dependence and high motivation to quit heroin’ subgroup, the ‘More complex clinical presentation, low risk of alcohol dependence and high motivation to quit heroin’ subgroup, the ‘More complex clinical presentation, high risk of alcohol dependence and high motivation to quit heroin’ subgroup, and lastly the ‘More complex clinical presentation, low/moderate alcohol dependence and low motivation to quit heroin’ subgroup.

All study participants were individuals with OUD seeking a new episode of OAT in the PRAISe trial, with notable heterogeneity among patients. Understanding these factors that differ significantly between subgroups is important to help characterise subgroup phenotypes. The ‘Less complex clinical presentation, low risk of alcohol dependence and high motivation to quit heroin’ subgroup is strongly characterised by an array of indicators; no service users reported ever having injected heroin, with less polydrug use, better mental health etc., factors distinctly representative of service users with less severe clinical presentation of OUD. The other three subgroups of ‘More Complex’ clinical presentation are less distinguishable, differing principally in terms of key individual indicators: motivation to quit heroin use, risk of alcohol dependence and ‘impulsivity’.

It should also be noted that a higher proportion of participants from ethnic minority backgrounds were represented in the less clinically complex group. One possible explanation is differences in routes of drug administration. Participants in this group may have been less likely to inject heroin and more likely to smoke it, which placed them within this participant category. The UK Drug Policy Commission reported in 2010 that Asian individuals who used drugs are more likely to smoke heroin, whereas individuals in White communities are more likely to inject [44]. However, caution is needed when interpreting the representation of individuals from ethnic minority backgrounds in treatment data. Overall, people who describe themselves as White are overrepresented in treatment services, accounting for around 90% of those accessing treatment in 2021–22. This includes 90% of those accessing opiate treatment (NDTMS Data 2016–2017 and 2023–2024 [45]). Importantly, the relatively low representation of people from minority ethnic groups in treatment data should not be interpreted as a lack of need within these communities. Instead, it may reflect barriers that people from ethnic minority backgrounds face when accessing services. Treatment providers should therefore review how effectively they are meeting the needs of ethnic communities. Organisational and workforce development programmes should also consider how services can better respond to the needs of different ethnic groups.

This study further explored the impact of belonging to each of the four characteristic groupings on the effectiveness of CM (targeted at either abstinence or attendance) on abstinence within the PRAISe trial [18]. We found evidence that CM targeted at attendance may be more effective in encouraging heroin abstinence in certain subgroups of patients receiving treatment for OUD; CM targeting attendance was determined effective in encouraging abstinence for the ‘Less complex clinical presentation, low risk of alcohol dependence and high motivation to quit heroin’ subgroup, and to a lesser extent the ‘More complex clinical presentation, low risk of alcohol dependence and high motivation to quit heroin’ subgroup, and not effective for the other two subgroups (findings collectively presented in the primary PRAISe study; CM targeting attendance is effective for the overall service user population [18]). This highlights the importance of patient heterogeneity in individual patient response to CM treatment. Individual characteristics used to determine the subgroups are evidenced/theorised to impact CM effectiveness [19], and therefore classes based upon heterogeneity across these characteristics are likely to have differing response to CM.

Following this, the CM intervention targeting attendance was potentially more effective for the grouping characterised by less complex clinical presentation. This conclusion has mixed support from previous literature. There is evidence that treatment in general has lesser benefit for patients with more complex clinical need; high levels of polydrug use is associated with higher levels of primary drug use in treatment for heroin [46] and injection use is predictive of higher levels of drug use through the first 3‐months of methadone maintenance treatment [47]. For CM treatment specifically, self‐reported past month benzodiazepine use is associated with blunted response to CM [48]. However, other individual characteristics indicative of more complex needs such as more previous treatment attempts [49] are associated with enhanced CM treatment outcomes.

This ambiguity regarding the impact of characteristics representing complex clinical presentation on the effectiveness of CM highlights the importance of looking at characteristics in the wider context of subgroups, to obtain a more comprehensive understanding of the impact of overall patient heterogeneity. Notably, nuances between the ‘More Complex’ clinical presentation subgroups, such as the ‘More complex clinical presentation, low risk of alcohol dependence and high motivation to quit heroin’ subgroup reporting slightly lower benzodiazepine use with a similar or greater number of previous treatment attempts, support that CM targeting attendance may be more effective for this ‘More Complex’ subgroup only. The impact of self‐reported past month benzodiazepine use on diminishing response to CM may also be greater than the impact of more previous treatment attempts on enhancing response to CM, and therefore when these characteristics are observed together, the service user would likely have diminished response to CM.

Other key difference factors between characteristic groupings, such as motivation to quit heroin use and impulsivity, which characterise the ‘More complex clinical presentation, low/moderate risk of alcohol dependence and low motivation to quit heroin’ subgroup, are also likely to impact CM effectiveness. Though previous studies have found that ‘readiness for change’ scores and motivation to quit are not associated with abstinence outcomes [50, 51], a process evaluation of the PRAISe trial suggested that there was a prevalent view among trial participants that CM was unlikely to promote abstinence unless individuals already felt a personal readiness for change [52]. This is represented in the findings of this study; CM targeting attendance was effective for subgroups with higher motivation to quit heroin use (excepting the group with high motivation in addition to high alcohol dependence). Additionally, higher delay discounting, or ‘impulsivity’ at treatment entry is also more commonly associated with poorer treatment outcomes [53]. Again, through looking at these individual characteristics in the context of subgroups, the simultaneous effect of both impulsivity and low motivation to quit heroin use may be evidenced to collectively diminish the effectiveness of CM.

Reported risk of alcohol dependence is also a key differentiator between characteristic groupings. CM targeting attendance was ineffective for the ‘More complex clinical presentation, high risk of alcohol dependence and high motivation to quit heroin’ subgroup, but effective for the ‘More complex clinical presentation, low risk of alcohol dependence and high motivation to quit heroin’ subgroup, suggesting alcohol dependence diminishes impact of CM targeting attendance. Alcohol use is also an established risk factor for non‐compliance with addiction treatment among OAT participants [54]. However, for CM specifically, current alcohol dependence is not related to treatment outcomes [49]. It should be noted here that there is a significant difference in the number of participants populating the two comparable subgroups, which impacts the power with which inferences can be drawn of the effectiveness of CM. The larger subgroup (‘with low risk of alcohol dependence’) has a much smaller confidence interval than the smaller subgroup (‘with high risk of alcohol dependence’), and therefore the difference determined in effectiveness could merely be due to power difference, a limitation of this study.

CM targeted at abstinence, which was not found to be effective in the overall PRAISe trial population, was also not significantly effective within any characteristic grouping. The lack of effectiveness of CM targeting abstinence in the overall PRAISe findings was regarded by the authors as a potential result of pragmatic adaptations made for the UK drug treatment service, including monitoring UDS results once‐weekly and having fixed incentives (instead of multiple UDS tests and escalating incentives [18]). This study's findings suggest that this pragmatic model of CM was ineffective independent of patient subgroups and heterogeneity across characteristics, therefore supporting that all patients may require a more resource‐intensive CM targeting abstinence, with less modifications, to realise treatment outcomes.

For CM targeted at attendance, however, this study supports the need for precision in addiction care and tailoring of treatment to the individual (or the characteristic grouping). This could be achieved by adjusting the dose of CM for those with more complex clinical problems, and those with poorer attendance rates given higher doses. A meta‐analysis of CM for treatment attendance found that more frequent monitoring and opportunities for receiving rewards for attendance were significantly associated with larger effect sizes [13]. Tailoring treatment to individual needs would be a more efficient use of staff time and resources. More research needs to be undertaken to assess whether CM targeted at attendance can successfully promote opioid abstinence.

The analysis focused on heroin abstinence as the main outcome, as this was the primary outcome for the PRAISe trial. However, we recognise that some service users may have other harm reduction goals. Future research might assess whether the latent classes differ in their response on a broader set of outcomes more aligned with harm reduction. This additional information could suggest further ways CM could be individualised and focused on enhancing goals that work better for the individual.

### 4.1 Strengths and Limitations

Though the study has a sufficient sample size (> 500) to identify an accurate class model [55], the sample size for moderation analysis was reduced within classes giving relatively large standard errors and confidence intervals. There was a difference in the number of participants who were excluded from the analysis due to missing all four UDS results at week 9, 10, 11 and 12 between trial arms; a higher proportion were excluded of those receiving CM targeting abstinence (16.6%) compared to TAU (13.0%) and CM targeting attendance (6.9%), likely due to assignment. All participants were asked to provide research UDS on weeks 9–12. Weekly ‘urine collection clinics’ were established by the research team at each clinic (participants were reimbursed for travel) to enable participants to provide UDS outside of keywork appointments. The pattern of missing data suggests participants receiving CM targeting abstinence were less likely to attend a weekly urine collection clinic than those receiving CM targeting attendance or TAU which might be due to an increased burden to provide UDS (they were providing weekly UDS as part of the CM Abstinence intervention) and/or a greater likelihood of providing a positive urine although results of research UDS were not provided to them or their drug treatment service. We need to acknowledge there were no adjustments made for multiple testing, although this seems reasonable in an exploratory, secondary analysis of trial data. It should also be noted that while heroin use (primary outcome) was captured through an objective measure of UDS results, baseline characteristic data that was self‐reported could be biased (though self‐reported information about drug use has been shown to have high validity [15, 23]). Additionally, the indicator representing motivation to quit heroin use has not been psychometrically tested and validated, and was measured at baseline only and could be likely to change over time; there are unknowns about the reliability and construct and predictive validity of this scale. There was also a difference in dose and psychosocial interventions received between participants, which was not controlled for. In addition, there were limitations in the lack of ethnic and racial diversity in this trial population.

Using a 3‐step LCA procedure to account for uncertainty of class assignment was a strength of this study. However, the class solution should undergo external validation to demonstrate reproducibility in external datasets. The PRAISe trial population was recruited from 33 clinics across the United Kingdom, suggesting study robustness and good generalisability. Though, as is usual in trials, due to relevant and important inclusion/exclusion criteria (such as starting a new episode of OAT instead of undergoing continuous OAT), participants were probably more homogeneous than a general clinic population. It would be interesting to repeat a similar analysis in a more heterogeneous clinic‐based population.

This study is based on data collected between 2012 and 2015. While the overall profile of people entering treatment for OUD in the United Kingdom has remained broadly stable over the past decade—particularly in terms of gender, age distribution, history of injecting, and experience of homelessness [2]—there have been important changes in patterns of drug use, clinical complexity, service delivery and policy. Heroin remains the most used opioid among people in treatment [1]. However, the opioid drug market and patterns of use have evolved. There has been a rise in the availability and use of synthetic opioids, and people entering treatment are increasingly medically complex, often presenting with co‐occurring mental health conditions and significant physical health comorbidities. In addition, heroin use is increasingly accompanied by crack cocaine use. That said, in the trial population studied between 2012 and 2015, a high majority of participants were using both heroin and crack cocaine. Treatment provision has also changed. One key development has been the introduction of the long‐acting injectable buprenorphine formulation, Buvidal, which offers an alternative to daily oral supervised consumption and may improve adherence and reduce diversion. In addition, the COVID pandemic prompted changes in OAT delivery. In April 2020, due to concerns around the risk of transmission, guidance from the Department of Health and Social Care permitted patients to take home a two‐week supply of OAT for unsupervised consumption. However, following concerns about increased methadone‐related deaths between April and September 2020, in 2021 supervised consumption was reinstated. Since then, supervision has been applied more selectively, focusing on individuals considered at higher risk of missed doses [56]. Treatment funding has also fluctuated over this period. Investment in drug treatment services declined after 2015, contributing to pressures on service capacity. However, with renewed policy attention with the Independent Review of Drugs [1], recommending national strategies aimed at strengthening treatment and recovery systems, funding has increased. In summary, although the demographic profile of people entering OUD treatment has remained relatively consistent, the past decade has seen notable shifts in new synthetic opioid drugs, drug use patterns, increasing clinical complexity, innovations in pharmacological treatment, changes to supervised consumption and changes in funding and policy priorities.

## 5 Conclusion

This study demonstrates the importance of understanding the heterogeneity within the OUD population, and that a more targeted treatment approach may be beneficial. CM targeted at attendance is potentially more effective in encouraging heroin abstinence for a subgroup of the OUD population with less complex needs and high motivation to quit heroin use, and more complex needs, high motivation to quit heroin use and low risk of alcohol dependence. Further studies are needed to consolidate this conclusion and lead to a better understanding of which sub‐populations might most benefit from receiving CM (targeted at abstinence or attendance). This work has the potential to inform future clinical guidelines.

## Author Contributions

Each author certifies that their contribution to this work meets the standards of the International Committee of Medical Journal Editors.

## Funding

The PRAISe trial was funded by the National Institute for Health and Care Research (NIHR) under its Programme Grants for Applied Research Programme (grant reference number RP‐PG‐0707‐10149) and has been delivered through the National Institute for Health and Care Research (NIHR) Biomedical Research Centre (BRC): Maudsley. L.M. was funded by a NIHR pre‐doctoral fellowship Ref: NIHR303472. K.G. and J.S. are part‐funded by the National Institute for Health and Care Research (NIHR) Biomedical Research Centre (BRC): Maudsley. K.G. and J.S. were supported by the National Institute for Health and Care Research (NIHR) Applied Research Collaboration South London (NIHR ARC South London) at King’s College Hospital NHS Foundation Trust. The views expressed are those of the authors and not necessarily those of the NIHR or the Department of Health and Social Care.

## Ethics Statement

The Positive Reinforcement targeting Abstinence In Substance misusE (PRAISE) trial was reviewed at the NRES Committee South East Coast Surrey (12/LO/0910) and received a favourable opinion on 25 July 2012.

## Conflicts of Interest

K.G. reports funding from the NIHR Biomedical Research Council, NIHR Applied Research Collaboration, other NIHR funding streams, Wellcome Trust, National Institutes of Health (US) and the Medical Research Council. T.W. declares no conflicts of interest. J.S. is a clinician and academic who has chaired committees for NICE and for Department of Health and National Treatment Agency, tasked with reviewing evidence of therapeutic interventions relevant to the addictions treatment field and from which reports have recommended contingency management interventions in the treatment of individuals in treatment for opioid use disorders. Through the university, he has also worked with pharmaceutical and technology companies, some of whom have provided the university with project grant support and/or consultancy payments, but these do not have any connection to the topic area considered in this paper. J.S. is also currently working with tech companies (CMI) to explore integration of CM strategies into app or device‐delivered therapeutic interventions, but these are not applicable to the study which generated the data involved with this paper. N.M. has received, through her university, King's College London, research funding from Mundipharma Research Ltd. (pharmaceutical company that produces a naloxone nasal spray). She is currently working with a tech company (CMI) to develop smart phone Apps to deliver digital contingency management and appointment reminders.

## Data Availability

The data that support the findings of this study are available on request from the corresponding author. The data are not publicly available due to privacy or ethical restrictions.
